# A Metabolic Insight Into the Neuroprotective Effect of Jin-Mai-Tong (JMT) Decoction on Diabetic Rats With Peripheral Neuropathy Using Untargeted Metabolomics Strategy

**DOI:** 10.3389/fphar.2020.00221

**Published:** 2020-03-05

**Authors:** Qian Zhang, Wei Song, Xiaochun Liang, Jun Xie, Yue Shi, Xiaohu Shi, Bintao Qiu, Xiuting Chen

**Affiliations:** ^1^Department of Traditional Chinese Medicine, Peking Union Medical College Hospital, Peking Union Medical College, Chinese Academy of Medical Sciences, Beijing, China; ^2^Medical Research Center, Peking Union Medical College Hospital, Peking Union Medical College, Chinese Academy of Medical Sciences, Beijing, China

**Keywords:** diabetic peripheral neuropathy, Traditional Chinese Medicine, Jin-Mai-Tong decoction, metabolomics, diabetes

## Abstract

Jin-Mai-Tong (JMT) decoction is a traditional Chinese compound prescription for treating diabetic peripheral neuropathy (DPN). The aim of this study is to investigate the neuroprotective effect of JMT decoction on diabetic rats with peripheral neuropathy and to elucidate the potential mechanism based on a metabolomics approach. Sprague-Dawley (SD) rats were randomly divided into four groups: control group, Streptozotocin (STZ) induced model group, JMT low dose (JMT-L) treated group and JMT high dose (JMT-H) treated group. After 12 weeks of treatment, behavioral changes, small fiber loss, and histopathological damages of sciatic nerves were estimated. Serum samples were collected for untargeted metabolomics analysis based on UPLC/QTOF-MS and multivariate statistics. As a result, JMT treatment at two dosages (13.9 and 27.8 g/kg⋅d) evidently improved the mechanical pain threshold (*P* < 0.05), increased the intraepidermal nerve fiber density (IENFD) and subepidermal nerve fiber density (SNFD) (*P* < 0.05), and renovated the demyelination and axonal atrophy of sciatic nerves on DPN rats. Furthermore, metabolomics study revealed that the serum metabolic profiles altered significantly among the control group and the STZ-induced model group. A total of 21 metabolites were identified as potential biomarkers related to the therapeutic effect of JMT decoction. Among them, 16 biomarkers were found in both JMT-H and JMT-L treated groups, while the five others were specific to JMT-H group. These metabolites mainly involved in lipid metabolism, tricarboxylic acid (TCA) cycle, amino acid metabolism, and so on. Besides, correlation analysis indicated that both mechanical pain threshold and distal nerve fiber density were negatively correlated with the serum levels of metabolites from lipid metabolism and TCA cycle. In conclusion, the results demonstrated that JMT decoction has an obvious protective effect against DPN, which could be mediated via ameliorating the metabolic disorders in diabetic rats with peripheral neuropathy.

## Introduction

Diabetic peripheral neuropathy (DPN) is a common and long-term complication of both type 1 and type 2 diabetes, which not only leads to neuropathic pain (Pop-Busui et al., 2017), but also increases the risk factor of foot ulcer and amputation (Holman et al., 2012; Boulton, 2013; Vas and Edmonds, 2016). DPN affects more than 50% of diabetic patients (Feldman et al., 2017) and has been supposed to be an independent predictor for all-cause and diabetes-related mortality (Hsu et al., 2012). Pathophysiological processes of DPN include enhanced activity of the polyol pathway, protein kinase C activation, increased advanced glycation end-products, oxidative stress, and so on (Feldman et al., 2017). Actually, chronic hyperglycemia leads to downstream multiple metabolic dysfunctions. Recent attentions have been focused on the global status of metabolism under hyperglycemia condition (Filla and Edwards, 2016; Freeman et al., 2016; Feldman et al., 2017). For instance, glucose and polyol intermediates in the peripheral nervous tissues of STZ-induced diabetic rats were significantly increased when compared to healthy rats, and the sciatic nerves of diabetic rats exhibited striking upregulation of mitochondrial oxidative phosphorylation and disorder of lipid metabolism (Freeman et al., 2016). Diabetes also induced extensive changes of the cortical myelin at metabolic level (Cermenati et al., 2017). Despite extensive efforts into treatment of DPN, drugs targeting casual mechanism such as antioxidants, aldose reductase inhibitors, and growth factors still have limited efficacy in clinical practices (Farmer et al., 2012). At present, treatment targeting single pathway seems to be insufficient against DPN. Additional novel treatment strategies which are beneficial to the whole metabolic profile need to be explored.

Traditional Chinese Medicine (TCM) is a complex mixture of treatments that have been used for preventing diabetes and its complications since 2000 years ago in China. JMT decoction, which composed of 12 different drug materials, has been used to treat DPN in clinical practices for more than 20 years in Peking Union Medical College Hospital (PUMCH). Our previous clinical randomized controlled study have proved that JMT decoction alleviated the DPN clinical symptoms and improved nerve conductive velocity of DPN patients (Liang et al., 1999). We found that JMT decoction played the therapeutic role in reducing oxidative stress (Yin et al., 2015), increasing neurotrophic factors (Shi et al., 2013), inhibiting high glucose induced apoptosis of Schwann cells (Wang et al., 2013) and regulating autophagy *in vivo* and *in vitro* (Qu et al., 2016). Besides, our recent research found that JMT decoction could increase gene and protein expression of insulin-like growth factor 1 (IGF-1) and the insulin like growth factor 1 receptor (IGF-1R), as well as regulate the expression of nerve remyelination genes P0 and PMP22 in sciatic nerves of diabetic rats (Song et al., 2019). However, TCM has been characterized as a multi-target therapy and JMT decoction could be the same. It remains unknown that whether JMT decoction could protect against DPN via regulating metabolic disorder.

In the last decade, the development of metabolomics technology provided new strategy and comprehensive insight to assess the global alteration of the study objects at metabolic level. Small molecules in biological samples could be detected and semi-quantitative unbiasedly using untargeted metabolomics approach, which is a powerful tool for illustrating the inherent metabolic changes and elucidating the pharmacological mechanism of drugs on DPN (Filla and Edwards, 2016). In the present study, untargeted metabolomics based on UPLC/QTOF-MS system and multivariate statistical analysis was established to investigate the effect of JMT decoction on metabolic alteration related to chronic hyperglycemia in DPN rat model. Prominent metabolites that may be related to the therapeutic effects of JMT decoction were screened out using multivariate analysis. The metabolic pathway was also enriched based on KEGG database. Overall, this research provided a new insight to elucidate the potential therapeutic effects of JMT decoction on DPN at metabolic level.

## Materials and Methods

### Chemicals and Reagents

Acetonitrile of HPLC-grade was acquired from Merck KGaA (Merck, Darmstadt, Germany). Deionized water was prepared using a Millipore water purification system (Millipore, Bedford, MA, United States). Formic acid and Streptozotocin (STZ) were procured from Sigma Aldrich (St. Louis, MO, United States). Rabbit polyclonal PGP 9.5 antibody was obtained from GeneTex (Irvine, CA, United States). Chloral hydrate from Macklin (Shanghai, China) was used to anesthetize rats. Paraformaldehyde were purchased from Coolaber (Beijing, China).

### Preparation of JMT Decoction

Jin-Mai-Tong (JMT) decoction composed of 12 kinds of drug materials, including Semen Cuscutae (the seeds of *Cuscuta chinensis* Lam.), Fructus Ligustri Iucidi (the seeds of *Ligustrum lucidum* W. T. Ait.), Herba Ecliptae [the herb of *Eclipta prostrata* (L.) L.], Herba Prunella Vulgaris (the herb of *Prunella vulgaris* L.), Semen Litchi (the seeds of *Litchi chinensis* Sonn.), Scorpio (*Buthus martensii* K.), Ramulus Cinnamoml [the tender stem of *Cinnamomum cassia* (L.) J. Presl.], Rhizoma Corydalis (the rhizoma of *Corydalis yanhusuo* W. T. Wang), Semen Persicae (the seeds of *Prunus persica* L.), Senmen Cassiae [the seeds of *Senna obtusifolia* (L.) H. S. Irwin & Barneby], Radix et Rhizoma Asari (the radix and rhizoma of *Asarum heterotropiodes* F. Schmidt), and Hirudo (*Hirudo nipponica* W.), with a fixed ratio of 10: 10: 10: 10: 30: 3: 10: 10: 10: 30: 3: 3 as our previously report (Song et al., 2019). All the crude drugs were purchased from Tong Ren Tang Lit. Corp (Beijing, China) and authenticated by Prof. X.C. Liang (Peking Union Medical College Hospital, Beijing, China) based on the botanical traits recorded in the Chinese Flora^1^. The batch number and other detailed information of each drug material were given in Supplementary Table S1 and Supplementary Figure S1. The voucher specimens were deposited at the Department of Traditional Chinese Medicine, Peking Union Medical College Hospital, Beijing, China.

For preparation of the JMT decoction, the mixed crude drugs were soaked with stilled water at room temperature (25°C) for 2 h. For the first decoction, the drugs were refluxed with 10-fold of water (1:10, *w*/*v*) for 1.5 h before filtered. For the second decoction, the drug residues were refluxed with eightfold of water (1:8, *w*/*v*) before filtered. The two decoctions were then mixed together and concentrated in vacuum. The concentrated decoction was freeze-dried with an extraction yield of 19%. Then the JMT extract was stored under −80°C and well suspended in water before use.

### Chemical Analysis of JMT Decoction

The freeze-dried powders of JMT decoction water extract (20 mg) was dissolved in 10 mL of distilled water and then filtered with a 0.22 μm membrane before analysis. The chemical profile of JMT decoction was performed by HPLC/Triple-TOF MS (Sciex, United States) equipped with an ESI source. Separation was achieved on an HSS T3 column (100 mm × 2.1 mm, 1.7 μm; Waters). Mobile phase A was water containing 0.1% formic acid and mobile phase B was 0.1% formic acid in acetonitrile. A gradient elution program was applied (0–2 min, 5–20% B; 2–12 min, 20–40% B; 12–22 min, 40–65% B; 22–25 min, 65–90% B). The column temperature was maintained at 45°C and the flow rate was 0.2 mL/min. MS data was acquired in negative ion mode at a range of m/z 50–1500. For the mass spectrometer system, the curtain gas, gas 1 and gas 2 were set to 0.241, 0.276, and 0.276 MPa, respectively. The electrospray ion source temperature and spray voltage were 450°C and 4500 V, respectively. Following this approach, a total of 55 compounds were putatively identified by comparing their high-resolution MS data with reported data, all of which had been identified from a 1:1-combined methanol extract and water extract of JMT decoction in our previous study (Song et al., 2019). The base peak chromatography and detailed information of the 55 compounds were given in Supplementary Tables S1, S2 and Supplementary Figure S2.

### Animals and Treatment

This study was carried out in accordance with the principles of the Basel Declaration and recommendations of the Guide for the Care and Use of Laboratory Animals of the National Institutes of Health. The protocol was approved by the Experimental Animal Ethics Committee of Peking Union Medical College Hospital (the application number is XHDW-2018-009). Male Sprague-Dawley (SD) rats (6 weeks old) were obtained from Vital River Laboratory Animal Technology Co., Ltd. (Beijing, China, certificate No. SCXK Jing 2011-0004) and kept in the Experimental Animal Center (specific pathogen-free level) of the Peking Union Medical College Hospital (Beijing, China) with *ad libitum* access to food and water. Animals were maintained on a 12:12 h light-dark cycle. Diabetic rats were induced by single intraperitoneal injection of STZ in 0.9% sodium chloride at the dose of 55 mg/kg after an overnight fast (Al-awar et al., 2016; Yan et al., 2018). Hyperglycemia was confirmed 3 days after STZ injection and rats with blood glucose level > 16.7 mmol/L were considered as diabetic rats (Yang et al., 2015) and then randomly divided into three groups (STZ group, STZ + JMT-L group, and STZ + JMT-H group, *n* = 6 per group). Meanwhile, age-matched control rats were intraperitoneally injected with normal saline of same volume (Control group, *n* = 6 per group). Blood glucose was measured from the tail tip using the Accu-Chek Active Blood Glucose Meter and the matched test strips (glucose dehydrogenase methods) (Roche Diabetes Care Inc., Ireland).

Rats in STZ + JMT-L group and STZ + JMT-H group were orally administrated with JMT decoction at the dosage of 13.9 g/kg (equal to clinical dose, calculated by weight of crude drugs) and 27.8g/kg, respectively, once per day and for consecutive 12 weeks after diabetes model was established. Rats in STZ group and control group were administrated with equal volume of distilled water (5 mL) once per day. Body weight and tail tip blood glucose levels of all rats were measured every 4 weeks.

### Von Frey Sensory Testing

After 12 weeks of treatment, mechanical sensitivity to noxious stimuli was assessed using an aesthesiometer of the Von Frey Pain Measurement Instrument (IITC Life Science Inc., Woodland Hills, CA, United States) (Choi et al., 2014; Yerra et al., 2018). As described earlier, after the rats adapted the surrounding environment, the hind paw was stimulated by a probe of the Von Frey instrument (Song et al., 2019). Mechanical pain threshold was measured as the force at which paw withdrawal was observed and recorded in grams. Each rat was measured 3 times on bilateral hind paws with a 5 min interval between consecutive stimuli. The mechanical pain threshold of rats was considered as the average measurements.

### Observation of Morphological Changes in Sciatic Nerve

At the end of the experiment, all rats sacrificed by intraperitoneally injection of 10% chloral hydrate (3 mL/Kg) for deep anesthesia. After blood collection, the left sciatic nerve (2–3 mm) were isolated and fixed in 4% paraformaldehyde (PFA) in 4°C overnight. Sciatic nerve was paraffin-embedded and cut both cross and longitudinal section (4 μm). Routine histopathological procedure was conducted for Hematoxylin and eosin (H&E) staining for observing the pathologic structural changes and images were acquired by Aperio CS2 (Leica Biosystems, Richmond, IL, United States).

### Intraepidermal and Subepidermal Nerve Profile

Refer to the previous described methods, the distal nerve profiles were measured quantitatively (Lauria et al., 2005; Roy Chowdhury et al., 2012). In short, the plantar skin of the hind paw was fixed in 4% PFA overnight at 4°C. After passing through an ethanol gradient, xylene and paraffin, the samples were cut 50 μm sections and then treated with 0.5% Triton X-100 for 30 min. After incubation with goat serum, the sections were stained with an antibody to anti-protein gene product 9.5 (PGP 9.5) antibodies (1:200, GeneTex) overnight at 4°C, and then followed by incubation with a FITC conjugated goat anti-rabbit secondary antibody and finally mounted with anti-fade medium. The images were observed and collected by Nikon A1R confocal system (Nikon, Tokyo, Japan) (20×). Two observers blinded to the control or diabetic rats, independently counted the nerve fibers numbers in each section. Intraepidermal nerve fiber density (IENFD) was determined by the total number of PGP 9.5 immuno-reactive nerve fibers at the dermo-epidermal junction divided by the length of the epidermal surface (nerve fibers/mm) (Lauria et al., 2005). Subepidermal nerve fiber density (SNFD) was determined by the number of nerve fibers at the papillary dermis (subepidermal nerve plexi) divided by the length of the epidermal surface (Roy Chowdhury et al., 2012). The epidermal surface of each section was calculated by Image J.

### Preparation of Serum Sample

At the end of the experiment, all rats were fasted for 12 h (free to drink water) and then sacrificed for serum collection under anesthesia. Blood were collected from the abdominal aorta. Each sample was standing at 4°C for 4 h, centrifuged at 3,000 rpm for 10 min, and the supernatant was collected and stored at −80°C until metabolic analysis. Prior to analysis, each serum sample was thawed at 4°C and an aliquot of 100 μL was transferred to a sterile siliconized 1.5 mL Eppendorf tube. After treated with 300 μL of cold methanol, the mixture was vortexed for 30 s and then standing at 4°C for 1 h to precipitate protein. After centrifuged at 13,000 g for 10 min, the supernatant was then collected and filtered through a 0.22 μm membrane before UPLC/Q-TOF MS analysis. Quality control (QC) sample was prepared by mixing equal volume (50 μL) of each test sample. The QC sample was injected 6 times at the beginning of the run in order to ensure the equilibration of the UPLC/Q-TOF MS system and then between every 6 samples among the run to ensure the consistency of the analysis.

### UPLC/QTOF-MS Conditions

Metabolic profiling was performed on a Xevo G2-XS Q-TOF mass spectrometer (Waters, Manchester, United Kingdom) which equipped with an ACQUITY UPLC system (Waters, MA, United States) via a Zpray^TM^ ESI source. Samples were separated on an Acquity UPLC HSS T3 column (1.8 μm, 2.1 × 100 mm) equipped with a VanGuard precolumn (1.8 μm, 2.1 × 5 mm) (Waters, MA, United States). The mobile phase consisted of acetonitrile (A) and water containing 0.1% formic acid (B), while a linear gradient elution program (0.0–18.0 min, 10%–90% A; 18–20.0 min, 90–100% A) was applied for favorable separation. The column temperature was 40°C. The flow rate was set at 0.4 mL⋅min-1. An aliquot of 0.2 mL of the sample solution was injected for analysis. The Q-TOF mass spectrometer was operated in both negative and positive ion mode in centroid mode using MSE function, while low energy was 5 eV and high energy ramp from 20 to 40 eV. Other parameters were as follows: capillary voltage, 3.5 kV (negative)/3.0 kV (positive); cone voltage, 25 V; source temperature, 120°C; desolvation temperature, 350°C; desolvation gas flow rate, 800 L⋅h^–1^; cone gas flow rate, 10 L⋅h^–1^. The MS data were collected in centroid mode over an m/z range of 50–1000 Da with scan time of 0.1 s. Accurate mass was maintained by introducing the LockSpray interface of leucine-enkephalin (m/z 556.2771 in ESI+, m/z 554.2615 in ESI-) at concentration of 200 ng/μL with rate of 10 μL/min. Data were acquired in centroid mode using the MassLynx software (Waters, MA, United States).

### Data Processing and Statistical Analysis

Raw data preprocession and multivariate analysis for metabolomics study were performed using MetaboAnalyst 4.0^2^. After a programmed processing, the resulting three dimensional data matrixes contained sample description, normalized peak areas, and the retention time-m/z pairs were proceed for multivariate analysis. Principal component analysis (PCA) was used to display the overall differences. Partial least-squared discrimination analysis (PLSDA) and orthogonal projection to latent structure-discriminate analysis (OPLS-DA) were used to verify the model and to explore the different metabolites between groups. Metabolites selected as biomarker candidates for further statistical analysis were identified on the basis of variable importance in the projection (VIP) ≥ 1 from the 10-fold cross-validated OPLS-DA model, which was validated at a univariate level with adjusted *P* < 0.05.

Data were presented as the mean ± standard deviation for continuous variables. Student’s *t*-test was used for comparison between two groups. One-way ANOVA analysis was performed for multiple comparison. When ANOVA showed statistical significance, *post hoc* analysis was done to further comparison between two groups. *P* < 0.05 indicated a statistical significance. Spearman rank correlation analysis was done and scatter plot was drawn to show the relationship between two variates by Graph Pad Prism, Version 8.0.

### Metabolite Identification and Metabolic Pathway Analysis

As described in our recent study (Liu et al., 2019), metabolites were identified according to their exact molecular weight and the MS/MS fragmentation pattern by comparison with those in the online HMDB database^3^ (Wishart et al., 2018), KEGG database^4^ (Kanehisa and Goto, 2000), and METLIN (Sud et al., 2007). The mass error was 10 ppm for MS^1^ and 15 ppm for MS^2^. MetaboAnalyst 4.0 was used for metabolic pathway analysis base on KEGG database (Smith et al., 2005).

## Results and Discussion

### Effect of JMT Decoction on Blood Glucose, Body Weight Loss, and Mechanical Pain Threshold of DPN Rats

**FIGURE 1:**
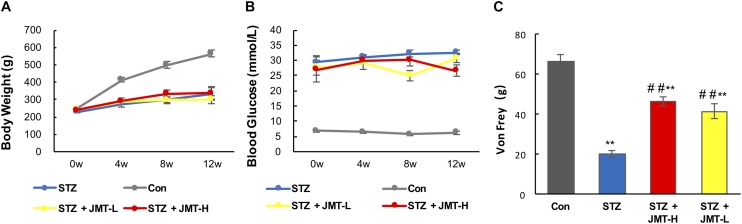
Effects1pc of JMT on body weight **(A)**, blood glucose **(B)**, and Von Frey sensory testing **(C)**. Data are group mean ± SEM (*n* = 6 per group). ^##^*P* < 0.01 vs. untreated diabetic rats; **P < 0.01 vs. control rats by Student’s *t-*test.

JMT decoctoin treatment at both high and low dosages did not affect the body weight of STZ-induced diabetic rats (Figure 1A, *P* > 0.05). As shown in Figure 1B, after treatment of 12 weeks, diabetic rats treated with JMT decoction at high dose exhibited lower blood glucose level than untreated diabetic rats (*P* < 0.01), while JMT decoction at low dose showed no significant hypoglycemic effect (*P* > 0.05). The slight decrease in blood glucose indicating a potential hypoglycemic effect of JMT decoction. However, the regulation of glucose metabolism was marginal as the blood glucose level of JMT-H treated diabetic rat was much higher than healthy normal rat. Additionally, the animal model employed in this study was type 1 diabetic rat model induced by STZ-injection, which is in absolute deficiency of insulin, so the potential hypoglycemic effect of JMT decoction observed here might not be insufficient to demonstrate its clinical significance. To further explore the hypoglycemic effect of JMT decoction and obtain more evidences for clinical practices, more experiments will be taken on type 2 diabetic animal model and by recruiting diabetic patients in the near future.

Mechanical pain threshold significantly decreased in diabetic model rats compared with those of the age-matched healthy rats (Figure 1C, *P* < 0.01). Oral administration of JMT decoction at low and high dose for 12 weeks significantly increased mechanical pain threshold compared to untreated diabetic rats (Figure 1C, *P* < 0.01). Besides, there was no significant difference on mechanical pain threshold between the two treatment groups using JMT decoction at high and low doses (Figure 1C, *P* > 0.05).

### JMT Decoction Improved Pathomorphological Changes of Sciatic Nerve in DPN Rats

**FIGURE 2:**
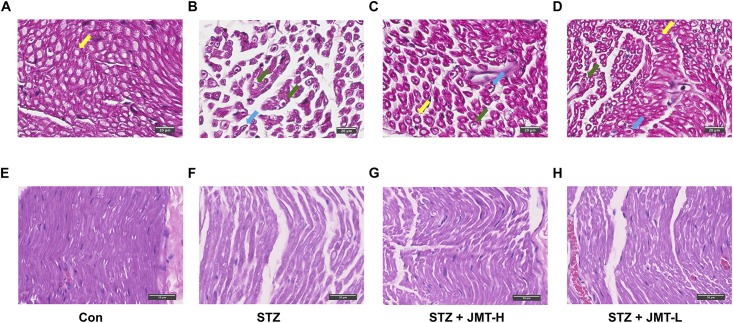
Histological examination of sciatic nerve in the cross-sectional **(A–D)** and longitudinal **(E–H)** view. Normal myelinated fibers of sciatic nerves were arranged in a dense and wrapped with myelin (yellow arrows). Sciatic nerves of DPN rats exhibited obvious vacuolar-like defects (blue arrows), demyelination changes and axonal shrinkage (green arrows). Magnification: 40×, scale bars: 20μm (cross section), and 50μm (longitudinal section).

**FIGURE 3:**
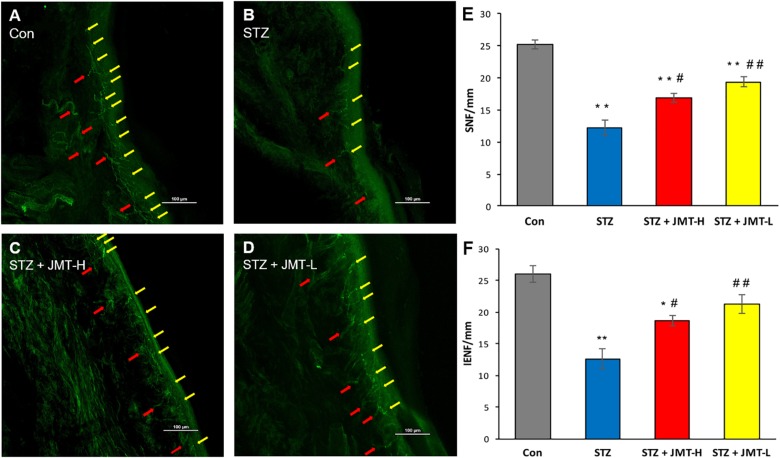
Foot skin nerve profiles detected by immunofluorescence assay. **(A–D)** Representative images of immunofluorescence staining of hind paw plantar skin nerves. The yellow arrows point PGP 9.5 immuno-reactive intraepidermal nerve fiber (IENF) and the red arrows point subepidermal nerve fiber (SNF). Magnification: 20×, scale bars: 100 μm; **(E)** SNFD (fibers/mm); **(F)** IENFD (fibers/mm). Data are group mean ± SEM (*n* = 3 per group). *P < 0.05 and **P < 0.01 vs. control group; ^#^*P* < 0.05 and ^##^*P* < 0.01 vs. untreated STZ group by ANOVA with Dunnett’s *post-hoc* test.

According to H&E staining (Figure 2), fibers of sciatic nerves in control group were arranged in a dense and wrapped with myelin sheath (Figures 2A,E). On the contrary, the fibers of rats in STZ model group were loose, and different in size (Figures 2B,F). The number and diameter of fibers were reduced in STZ-induced model group, depicted from both cross and longitudinal sections (Figures 2B,F). Nerve fibers of STZ-induced model rats tapered off to a point, indicating the shrinkage of the axon in the view of cross section (Figure 2B). The myelin sheath in STZ group exhibited increased vacuolar-like defects. And the lamellar spaces of myelin sheath were expanded or separated (Figure 2B). Visible signs of demyelination changes with axonal atrophy was evident in STZ group (Figures 2B,F). The nuclei of Schwann cells were elongated in shape in control group in the longitudinal view of sciatic nerve (Figure 2E). Compared to the normal rats, Schwann cells nuclei of rats in STZ model group were stained lighter, smaller in size and changed to a nearly round shape (Figure 3F). The staining of fibers in normal rats (Figures 2A,E) were deeper than that in STZ-induced model rats (Figures 2B,F), suggesting a loss of myelin in STZ model rats. These morphological damages of sciatic nerves were obviously reduced in JMT-L and JMT-H treated diabetic rats (Figures 2C,D,G,H). From the view of cross section (Figures 2C,D), compared with the rats in STZ group (Figure 2B), the rats in both JMT-L and JMT-H group exhibited decreased demyelination changes and alleviative axonal atrophy. The nerve fibers of rats in JMT-L and JMT-H groups were thicker and more numerous than those in STZ group. From the longitudinal section (Figures 2G,H), compared to STZ group (Figure 2F), nerve fibers in both JMT-L and JMT-H groups were arranged more orderly and stained deeper. In general, neurodegeneration changes, especially, demyelination with axonal atrophy in JMT-H and JMT-L treated diabetic rats were profoundly corrected.

### JMT Decoction Prevented Skin Nerve Fiber Loss of DPN Rats

Hind paw skin from STZ-induced 12-weeks diabetic rats showed a significant decrease in intraepidermal nerve fiber density (IENFD) and SNFD compared with that from the age-matched rats (Figure 3, *P* < 0.01). JMT-H and JMT-L administration significantly increased SNFD (Figure 3E, *P* < 0.05, and *P* < 0.01, respectively) and IENFD (Figure 3F, *P* < 0.05, and *P* < 0.01, respectively) compared with untreated diabetic rats. The difference of IENFD and SNFD between the JMT-L and JMT-H groups were not statistically significant (*P* > 0.05).

The peripheral nervous system, vulnerable to hyperglycemia, is composed of neurons and Schwann cells. There are unmyelinated axons known as C-fiber axons or small fibers and myelinated axons wrapped by myelin sheath in the peripheral nervous system. With the development of DPN, unmyelinated C-fibers occurs degeneration resulting in pain, allodynia, or sensation abnormalities. So DPN rodent models usually exhibit behavioral changes which could be evaluated by decreased mechanical pain threshold. As the course progresses, the DPN hallmarks are considered to be demyelination with axonal degeneration of myelinated fibers and small nerve fibers loss in both animal models and human (Feldman et al., 2017; Gonc̨alves et al., 2017). Skin nerve fiber loss, the current gold standard of small fiber neuropathy in both diabetic animal model and DPN patients (Biessels et al., 2014; Chen et al., 2015) can be quantified by IENFD and SNFD that was defined by the number of nerve marker PGP 9.5 positive fibers divided by the length of the epidermal surface. In our study, STZ-induced 12 weeks diabetic rats developed mechanical pain threshold decreasing and exhibited dramatic skin nerve fiber loss compared to the age-matched healthy rats. On the other hand, the myelin sheath made by Schwann cells forms a barrier between the extracellular milieu and the axonal compartment. The integrity of myelin sheath is essential for maintaining normal structure and function of peripheral nervous system (Feldman et al., 2017). As shown in Figures 2B,F, distinct demyelination of myelinated fibers with axonal atrophy in sciatic nerves of the diabetic rats were observed in histological examination, representing typical DPN pathologic changes. Besides, the loss of myelinated fibers and the nuclei changes of Schwann cells in sciatic nerve were also observed in diabetic rats. Collectively, the phenotype of STZ-induced diabetic rats in our study consisted of behavior abnormality, nerve fiber loss, and obvious damages of nerve structure, thus DPN rats model was considered to be established (Biessels et al., 2014). On this basis, our study further demonstrated that treatment with JMT decoction at low and high dose for 12 weeks significantly promoted the sensation recovery on DPN rats. It also protected both small fibers and myelinated fibers from hyperglycemia-induced nerve injuries, and resulted in a significant improvement on demyelination of myelinated fibers in sciatic nerve. Therefore, we verified that JMT decoction treatment markedly alleviated the functional and morphological changes in peripheral nerve of DPN rats.

### Metabolomics Analysis

#### Quality Assurance

To evaluate the repeatability of the established method, five extracted ions in positive ion mode (0.73_407.9660, 4.16_503.2578, 8.54_255.2217, 12.11_889.9594, and 16.20_743.5491, showed in form of retention time_m/z) and five extracted ions in negative ion mode (0.55_143.0938, 4.76_671.2985, 8.53_301.2971, 12.75_629.8071, and 16.89_955.4596) were selected from the quality control (QC) samples throughout the experiment. The precision of the peak area of the ten ions were evaluated. As shown in Supplementary Table S3, the RSDs of the peak area of the 10 selected ions were 2.53–11.29%, indicating good precision of the UPLC/QTOF-MS system. Besides, the correlation analysis of QC samples also showed satisfactory stability of the system with *R*^2^ ≥ 0.977 and 0.963 in positive and negative mode, respectively (Supplementary Figure S3). The result indicated that the metabolomics approach was repeatable and stable for the following research.

#### Multivariate Statistical Analysis

**FIGURE 4:**
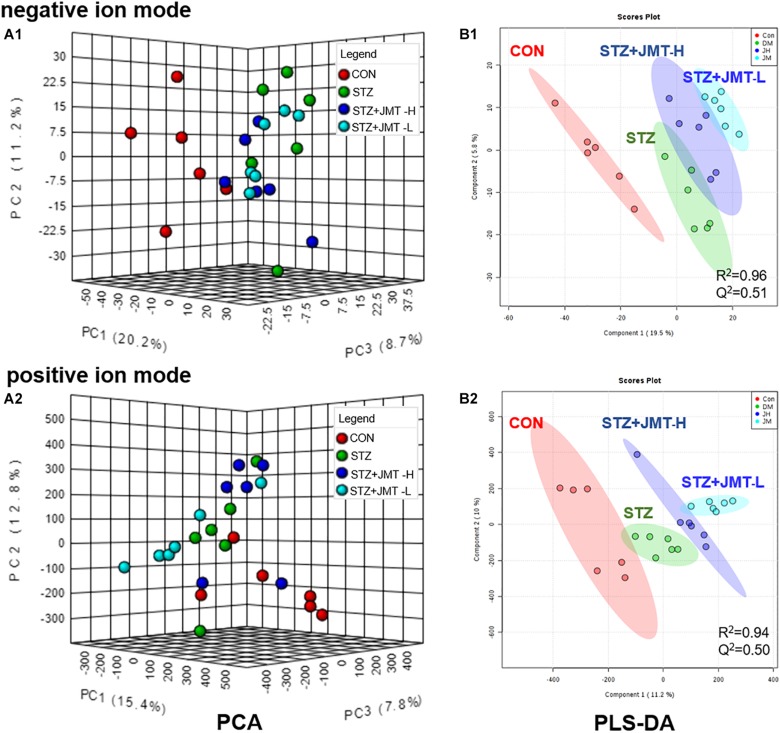
Discrimination of the serum metabolic profile among Control (CON), STZ, STZ + JMT-H, STZ + JMT-L rats. **(A1,A2)** The score plots of PCA in negative and positive ion modes, respectively. **(B1,B2)** The score plots of PLS-DA in negative and positive ion modes, respectively, *n* = 6 per group.

To obtain adequate information of metabolites, both positive and negative ion modes were applied to analyze the serum samples from control, STZ, STZ + JMT-H, and STZ + JMT-L groups. Principal component analysis (PCA) was first used to give an unsupervised and comprehensive view of the metabolic phenotype of all serum samples. As shown in Figure 4, the PCA score plot illustrated the distribution among the four groups in 3D space. Samples in CON and STZ groups were separated distinctly in both positive and negative ion modes, indicating that significant metabolic changes had appeared at 12 weeks after STZ induction. At the same time, the STZ + JMT-H and STZ + JMT-L groups had a relatively analogous metabolic phenotype, and seemed to display a restorable trend toward the control group, especially in negative ion mode and for the STZ + JMT-H group, which were basically consistent with the pathological differences. In addition, partial least squares-discriminant analysis (PLS-DA) was performed on all serum samples to maximize the class discrimination among the four groups, which possessed excellent goodness of fit (*R*^2^ ≥ 0.94) and acceptable predictive ability (*Q*^2^ ≥ 0.50). Distinct clustering of the control, STZ and STZ + JMT (H/L) groups was exhibited in both positive and negative ion modes, indicating that the group differences were more remarkable than individual differences (Figure 4). All above suggested the metabolic profiles were significantly altered in rat serum after STZ induction and JMT decoction treatment.

#### Screen and Identification of Potential Biomarkers

**FIGURE 5:**
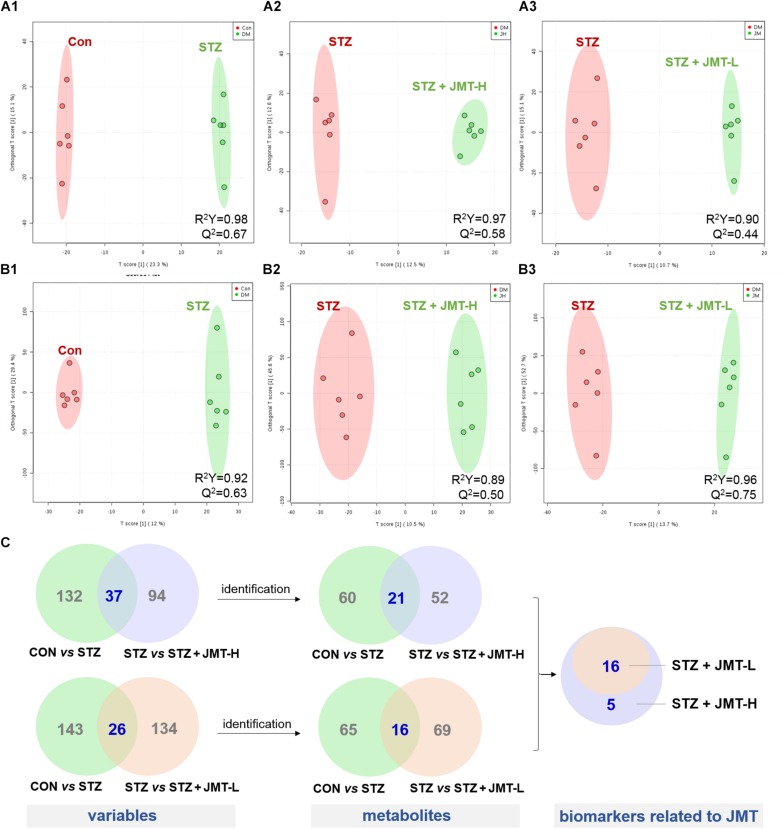
Screening of serum metabolic biomarkers regulated by JMT decoction administration. **(A,B)** The score plots of OPLS-DA between Control (CON) vs. STZ groups **(A1,B1)**, STZ vs. STZ + JMT-H groups **(A2,B2)**, and STZ vs. STZ + JMT-L groups **(A3,B3)** in negative and positive ion modes, respectively (*n* = 6 per group). **(C)** Venn diagram illustrated the distinct and overlapping metabolites that commonly changed in different groups. The serum levels of 16 metabolites were found to be regulated toward normal by JMT decoction administration at both low and high dosage, while another 5 metabolites were regulated just by JMT decoction at high dosage.

**FIGURE 6:**
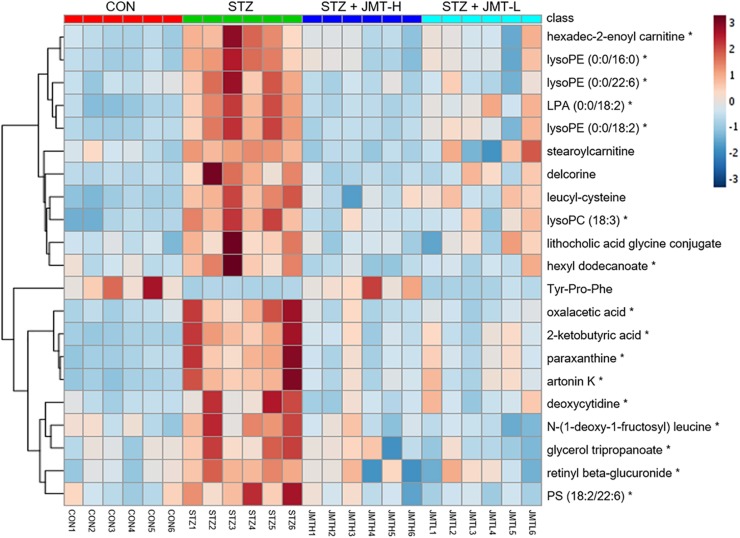
Heat map of 21 metabolic biomarkers in each sample. The serum levels of the 21 metabolites significantly renovated by administration of JMT decoction at high dosage (JMT-H) in control (CON), STZ, STZ + JMT-H, and STZ + JMT-L groups (*n* = 6 per group). *Metabolites also significantly renovated by administration of JMT decoction at low dosage (JMT-L).

Orthogonal partial least-squares discriminant analysis (OPLS-DA) was conducted to identify the significantly changed ions between control and STZ groups, STZ and STZ + JMT-H groups, as well as STZ and STZ + JMT-L groups. The OPLS-DA models were developed based on the maximized intergroup differences and minimized intragroup differences, and showed acceptable abilities for prediction and reliability with *R*^2^Y ≥ 0.89 and *Q*^2^ ≥ 0.44 (Figure 5). Then the variable importance in the projection (VIP)-plots were generated. In this study, variable ion with VIP value > 1 and *P* < 0.05 from an independent sample Student’s *t*-test was considered as potential biomarker that responsible for discrimination of the two groups. Following these criteria, 169, 131, and 160 variable ions were preliminarily screened out in control group vs. STZ group, STZ group vs. STZ + JMT-H group, and STZ group vs. STZ + JMT-L group, respectively. Then, the structures of these potential biomarkers were obtained by comparison of their high-resolution MS data and MS/MS fragments with those in free accessible databases as described in Materials and Methods section. Finally, 81 metabolites were identified from STZ groups, the concentrations of which were significantly altered when compared with control group. Among them, the serum levels of 21 metabolites (Table 1) were found to be regulated toward normal by JMT treatment at high dosage (VIP > 1 in OPLS-DA and *P* < 0.05). Besides, 16 of the 21 metabolites were also significantly modulated by JMT decoction treatment at low dosage. The relative contents of the 21 potential biomarkers in each sample were visualized using heat map analysis. As shown in Figure 6, 20 metabolites were up-regulated in STZ model group, while the other one was depressed. These metabolic disorders were corrected by JMT decoction in a great extent and could be contribute to the therapeutic effect of JMT decoction.

**TABLE 1:** 21 identified potential biomarkers regulated by Jin-Mai-Tong (JMT) decoction.

No.	Retention time (min)	Precursor ion form	Measured mass (Da)	Predicted mass (Da)	Mass Error (ppm)	Identification	Molecular formula	Trend in STZ^a^	Trend in STZ + JMT(H)^b^	Trend in STZ + JMT(L)^b^
1	0.51	[M + H]^+^	103.0392	103.0395	–2.91	2-Ketobutyric acid	C_4_H_6_O_3_	↑***	↓***	↓**
2	0.51	[M + H]^+^	181.0716	181.0726	–5.52	Paraxanthine	C_7_H_8_N_4_O_2_	↑**	↓***	↓*
3	0.52	[M-H] ^–^	233.0941	233.0960	–8.15	Leucyl-Cysteine	C_9_H_18_N_2_O_3_S	↑***	↓***	ns
4	0.53	[M + H]^+^	383.1162	383.1131	8.09	Artonin K	C_21_H_18_O_7_	↑**	↓***	↓*
5	0.56	[M + H]^+^	228.1003	228.0984	8.33	Deoxycytidine	C_9_H_13_N_3_O_4_	↑*	↓*	↓*
6	0.59	[M + H]^+^	133.0129	133.0137	–6.01	Oxalacetic acid	C_4_H_4_O_5_	↑***	↓**	↓**
7	8.68	[M + H]^+^	426.2027	426.2029	–0.47	Tyr-Pro-Phe	C_23_H_27_N_3_O_5_	↓*	↑**	ns
8	9.37	[M-H] ^–^	516.3083	516.3090	–1.36	LysoPC (18:3)	C_26_H_48_NO_7_P	↑***	↓***	↓*
9	9.92	[M + H]^+^	478.2934	478.2934	0.00	LysoPE (0:0/18:2)	C_23_H_44_NO_7_P	↑***	↓***	↓**
10	9.92	[M + H]^+^	435.2520	435.2512	1.84	LPA(0:0/18:2)	C_21_H_39_O_7_P	↑***	↓***	↓*
11	9.96	[M-H] −	478.2772	478.2805	–6.90	Delcorine	C26H41NO7	↑**	↓***	ns
12	9.97	[M + H]^+^	526.2971	526.2934	7.03	LysoPE (0:0/22:6)	C_27_H_44_NO_7_P	↑**	↓***	↓***
13	10.40	[M + H]^+^	398.3255	398.3270	–3.77	Hexadec-2-enoyl carnitine	C_23_H_43_NO_4_	↑**	↓***	↓**
14	10.40	[M + H]^+^	454.2922	454.2934	–2.64	LysoPE (0:0/16:0)	C_21_H_44_NO_7_P	↑***	↓***	↓*
15	10.84	[M-H]^–^	432.3100	432.3114	–3.24	Lithocholic acid glycine conjugate	C_26_H_43_NO_4_	↑**	↓**	ns
16	10.90	[M + H]^+^	294.1562	294.1553	3.06	N-(1-Deoxy-1-fructosyl) leucine	C_12_H_23_NO_7_	↑**	↓**	↓***
17	11.71	[M + H]^+^	428.3726	428.3740	–3.27	Stearoylcarnitine	C_25_H_49_NO_4_	↑**	↓***	ns
18	12.88	[M + H]^+^	261.1326	261.1338	–4.60	Glycerol tripropanoate	C_12_H_20_O_6_	↑**	↓*	↓***
19	12.90	[M + H]^+^	463.2680	463.2696	–3.45	Retinyl beta-glucuronide	C_26_H_38_O_7_	↑***	↓*	↓**
20	12.98	[M + H]^+^	832.5083	832.5129	–5.53	PS (18:2/22:6)	C_46_H_74_NO_10_P	↑**	↓**	↓***
21	14.53	[M + H]^+^	285.2771	285.2794	–8.06	Hexyl dodecanoate	C_18_H_36_O_2_	↑**	↓***	↓*

↑, upregulated; ↓, downregulated; *P < 0.05; **P < 0.01; ***P < 0.005; ns, no significant difference with P > 0.05. ^*a*^Compared with the control group. ^*b*^Compared with the STZ model group.

#### Metabolic Pathway Analysis and Biological Function of the Identified Biomarkers

**FIGURE 7:**
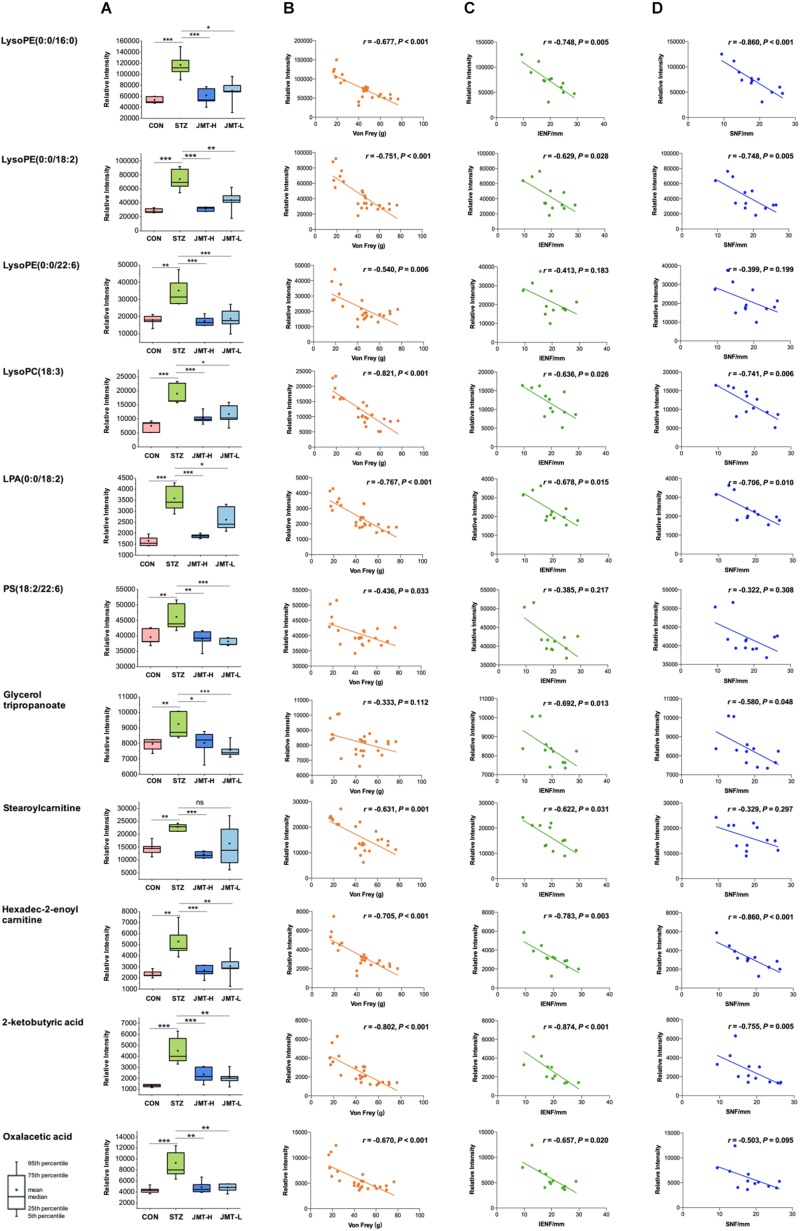
Serum levels of identified biomarkers in different group **(A)** and their correlations with DPN efficacy indicators (**B**, Von Frey; **C**, IENFD; **D**, SNFD) *P < 0.05, **P < 0.01, and ***P < 0.005. **(B)** Ten metabolites were significantly negatively correlated with Von Frey (*P* < 0.05). **(C)** Nine metabolites were significantly negatively correlated with IENFD (*P* < 0.05). **(D)** Seven metabolites were significantly negatively correlated with SNFD (*P* < 0.05).

To further reveal the therapeutic mechanism of JMT decoction in metabolic level, the 21 biomarkers in Table 1 were imported into MetaboAnalyst 4.0 for pathway topology analysis based on KEGG database (Ma et al., 2016). As shown in Supplementary Figure S4, JMT decoction mainly affected seven metabolic pathways, including glycerophospholipid metabolism, caffeine metabolism, glyoxylate and dicarboxylate metabolism, tricarboxylic acid (TCA) cycle, alanine, aspartate and glutamate metabolism, glycerolipid metabolism, and pyruvate metabolism. JMT decoction may affect these interrelated metabolic pathways to achieve the purpose of treating DPN. Besides, Spearman correlation analyses were conducted between DPN efficacy indicators (Von Frey sensory testing, IENFD, and SNFD) and the metabolic biomarkers involved in lipid metabolism, carnitine metabolism, and TCA cycle (Figure 7). Moreover, the biological functions of the biomarkers were interpreted as below.

Lipids are essential for maintaining the integrity of cell membranes and mitochondrial membranes, transmitting cell signals and regulating energy. In this study, significant accumulation of lipids was observed in serum of DPN rats. However, high-dose JMT administration brought back 9 intermediates related to lipid metabolism, including glycerophospholipid metabolites [lysoPE (0:0/16:0), lysoPE (0:0/18:2), lysoPE (0:0/22:6), lysoPC (18:3), LPA (0:0/18:2), PS (18:2/22:6)], triacylglycerols (glycerol tripropanoate), and carnitine (stearoylcarnitine and Hexadec-2-enoyl carnitine). Although hyperglycemia directly affects the peripheral nervous system, there are growing evidences showing that deranged lipid metabolism aggravates the onset and progression of DPN in both type 1 and type 2 diabetes (Wiggin et al., 2009; Smith and Singleton, 2013; Perez-Matos et al., 2017; Eid et al., 2019). Increased plasma triacylglycerols were found to be correlated with neuropathy progression, independent of glucose control in diabetic patients (Wiggin et al., 2009; Smith and Singleton, 2013). Alteration of phospholipids profiles were also observed in sciatic nerve and central nervous system of STZ-induced diabetic rodent models (Freeman et al., 2016; Cermenati et al., 2017; Sas et al., 2018). According to a detailed lipidomic analysis, abnormal lipid metabolism existed in myelin purified from sciatic nerves of diabetic rats and DPN was associated with accumulation of saturated fatty acids in myelin (Mitro et al., 2014). Consistent with previous report (Jiang et al., 2017), our results also indicated that some glycerophospholipids such as lysoPEs, lysoPCs, and LPA significantly increased in diabetic rats (Figure 7A). According to the correlative analyses (Figures 7B–D), six lipids intermediates were significantly and negatively correlated with Von Frey, while five lipids intermediates were negatively correlated with both IENFD and SNFD (*P* < 0.05). Particularly, JMT decoction administration remarkably down-regulated the serum level of these lipids intermediates toward normal. Stearoylcarnitine, a fatty ester lipid molecule, was found to be related to mitochondrial energy metabolism as accumulation of stearoylcarnitine could induce mitochondrial dysfunction (Aichler et al., 2017). Mitochondrial dysfunction may further lead to impaired axonal plasticity, axonal degeneration and nerve regeneration disorder in DPN (Edwards et al., 2010; Akude et al., 2011; Roy Chowdhury et al., 2011; Roy Chowdhury et al., 2012; Fernyhough, 2015). In our study, JMT decoction treatment at high dose restored the elevated level of stearoylcarnitine in serum of diabetic rats. These results indicated that the mechanism of JMT decoction relieving DPN was closely associated with the regulation of abnormal lipid metabolism.

Tricarboxylic acid (TCA) cycle, the main pathway of carbohydrate metabolism in mitochondria, was reported to be impaired in diabetes and its complications (Filla and Edwards, 2016; Wu et al., 2017; Mathew et al., 2019; Rojas et al., 2019). TCA cycle that is also related to mitochondrial function plays a central role in maintaining peripheral nervous function and structure. In our study, pathway analysis highlighted the TCA cycle in diabetic rats (Supplementary Figure S4). The abnormality of TCA cycle was also described in urine and sciatic nerve of STZ-induced diabetic mice (You et al., 2016; Rojas et al., 2019). From another integrated proteomics and metabolomics study (Freeman et al., 2016), six enzymes in TCA cycle were significantly increased in the sciatic nerves of diabetic rats. Among the metabolites, 2-Ketobutyric acid can be converted into propionyl-CoA and then subsequently methylmalonyl CoA, and thus enter the TCA cycle. Oxaloacetic acid is a four-carbon dicarboxylic acid appearing as an intermediate of the TCA cycle. Our study showed that the two metabolites of TCA cycle increased significantly in DPN rats compared with normal rats, and JMT treatment down-regulated the two metabolites toward normal. We further attempted to understand the link between sensation abnormalities or distal fiber loss with TCA cycle dysfunction. As shown in Figures 7B–D, the serum level of 2-Ketobutyric acid was negatively correlated with IENFD (*r* = −0.874, *P* < 0.001), SNFD (*r* = −0.775, *P* = 0.005) and mechanical pain threshold (*r* = −0.802, *P* < 0.001), respectively. Oxaloacetic acid was also negatively correlated with IENFD (*r* = −0.657, *P* = 0.020) and mechanical pain threshold (*r* = −0.670, *P* < 0.001), respectively. Thus, the effects of JMT decoction on regulation of TCA cycle may contribute to the recovery of nerve function and foot skin fiber loss of DPN rats.

In addition, JMT decoction treatment at high dosage also restored the serum levels of two amino acid residues [Tyr-Pro-Phe and N-(1-Deoxy-1-fructosyl) leucine] and one secondary bile acid (lithocholic acid glycine conjugate). The relationship between alteration in amino acid metabolism and peripheral nerve damage in diabetes also attracted attentions recently (Rojas et al., 2019). For example, some ranched-chain amino acids were reported to elevate in sciatic nerves of type 1 diabetic mice as early as 5 weeks post-STZ injection, suggesting that peripheral nervous system occurs disorders of amino acids metabolism under diabetes (Rojas et al., 2019). Bile acid are metabolized by enzymes derived from intestinal bacteria and linked to gut microbiota, lipid and carbohydrate metabolism (Jia et al., 2018). It has been proposed to be a key contributor to metabolic regulation so it will become a promising therapeutic targets for diabetes and its complications (Zaborskas and Cummings, 2018). Accordingly, the potential impact of JMT decoction on amino acid or bile acid metabolism may also be beneficial for the improvement of DPN.

Collectively, the metabolomics analysis revealed that JMT decoction treatment extensively regulated the metabolic disturbance in DPN rats which involved in multiple metabolic pathways. We also noticed a slight decrease of blood glucose in JMT-H group after the 12 weeks treatment. As is well known, tight glucose control in people with diabetes reduces the incidence of DPN (Pop-Busui et al., 2017), so the blood glucose reduction brought by JMT decoction might be beneficial to inhibit DPN development. However, the blood glucose level in JMT-L and JMT-H treatment groups was still far from the healthy control group. The reduction of blood glucose brought by JMT decoction was marginal, while the therapeutic effect on peripheral nervous dysfunction and structural changes were more obviously observed in JMT decoction treated DPN rats. Thus, we prefer to believe that the neuroprotective effect of JMT decoction is more likely to attribute to other metabolic pathways than to glucose regulated pathway. According to the serum metabolomics analysis, metabolites whose abnormal serum level called-back by JMT decoction were mainly distributed in lipid metabolism pathway, TCA cycle, amino acid metabolism pathway, and bile acid metabolism pathway. The overall improvement of multiple metabolic pathways arising by JMT decoction may mainly lead to the amelioration of DPN. Besides, the metabolomics approach was approved to be a useful tool to elucidate the therapeutic mechanism of JMT decoction on alleviating peripheral nervous injury related to diabetes.

Furthermore, the present research showed that JMT decoction treatment at higher and lower dosages exhibited metabolic-regulation effect to varying degrees. Although 5 more metabolic biomarkers were screened out in JMT-H group than in JMT-L group, there were still 16 biomarkers in common. According to our results, when compared with JMT decoction at low-dose (equivalent dose for adult human in clinic), JMT decoction at high-dose could modulated more metabolites and exhibited better therapeutic effect on metabolic disorder. Nevertheless, JMT decoction at both two doses effectively alleviated neuropathological changes and reduced the nerve fiber loss in DPN rats. However, it is a preliminary study using diabetic rodent model. The most appropriated dose of JMT decoction for clinical use still needs more consideration (such as long-term safety) and requires further investigations.

At last, it is challenging to establish the link between the neuroprotective effect of JMT decoction and the results of serum metabolomics. Nevertheless, some of the metabolic biomarkers found in our study have been reported to be associated with DPN. As discussed above, more than one metabolic pathways regulated by JMT decoction, such as lipid metabolism and TCA cycle, have been reported to be involved in DPN progression. Moreover, correlation analyses were conducted between metabolites involved in TCA cycle/lipid metabolism and three indicators of peripheral nerve injury (mechanical pain threshold, IENFD, and SNFD), the results of which showing that the serum levels of these typical metabolites were negatively correlated with these DPN-related indicators. In sum, those metabolic pathways regulated by JMT decoction were closely associated with DPN development, and some of the metabolic biomarkers were correlated to indicators of nerve injury, suggesting that the overall regulation on metabolic disorders could contribute to the neuroprotective effect of JMT decoction in DPN rats. Despite this, we should delve deeper to verify the metabolic differences on nerve tissues of DPN animals or serums of DPN volunteers, as well as perform further mechanism studies for better understanding the therapeutic effects of JMT decoction and applying it into the management of DPN.

## Conclusion

In this research, we found that JMT decoction treatment had a significant neuroprotective effect on DPN rats. Moreover, metabolomics study indicated remarkable alterations in serum metabolism. JMT decoction treatment significantly renovated abnormal metabolites which involved in lipid metabolism, TCA cycle, amino acid metabolism, and other metabolic pathways closely associated with DPN progression. Additionally, there was an obvious correlation between the indicators of peripheral nervous injuries and the serum levels of the metabolic biomarkers. The improvement of metabolic disorders could contribute to the neuroprotective effect of JMT decoction in DPN rats. In addition, this metabolomics-based approach was approved to be helpful in dissecting potential therapeutic effect and pharmacological mechanism of traditional medicine.

## Data Availability Statement

All datasets generated for this study are included in the article/Supplementary Material.

## Ethics Statement

The animal study was reviewed and approved by the Experimental Animal Ethics Committee of Peking Union Medical College Hospital.

## Author Contributions

XL contributed to the concept and design of the study and supervised this research. QZ and WS analyzed the data and wrote the manuscript. QZ, JX, YS, and XS performed the animal experiments. WS, BQ, and XC contributed to metabolomic analysis. All authors have read and approved the final manuscript.

## Conflict of Interest

The authors declare that the research was conducted in the absence of any commercial or financial relationships that could be construed as a potential conflict of interest.

## Footnotes

1 http://frps.iplant.cn/frps/Uncaria

2 https://www.metaboanalyst.ca

3 http://www.hmdb.ca

4 http://www.genome.jp/kegg/
