# Supplementary material for: A Metabolic Insight Into the Neuroprotective Effect of Jin-Mai-Tong (JMT) Decoction on Diabetic Rats With Peripheral Neuropathy Using Untargeted Metabolomics Strategy
Source: Front Pharmacol. 2020 Mar 5;11:221. doi: 10.3389/fphar.2020.00221 (PMC7066215; doi:10.3389/fphar.2020.00221)
Supplement: Supplementary file 1 [file Data_Sheet_1.pdf]

Supplementary Material

A Metabolic Insight Into the Neuroprotective Effect of Jin-Mai-Tong (JMT) Decoction on Diabetic Rats With Peripheral Neuropathy Using Untargeted Metabolomics Strategy

Qian Zhang[1†], Wei Song[2†], Xiaochun Liang[1*], Jun Xie[1], Yue Shi[1], Xiaohu Shi[1], Bintao Qiu[2], Xiuting Chen[2]

[1]Department of Traditional Chinese Medicine, Peking Union Medical College Hospital, Peking Union Medical College, Chinese Academy of Medical Science, Beijing, China

[2]Medical Research Center, Peking Union Medical College Hospital, Peking Union Medical College, Chinese Academy of Medical Science, Beijing, China

*** Correspondence:** Xiaochun Liang, Xiaochun_Liang@yeah.net

† These authors have contributed equally to this work

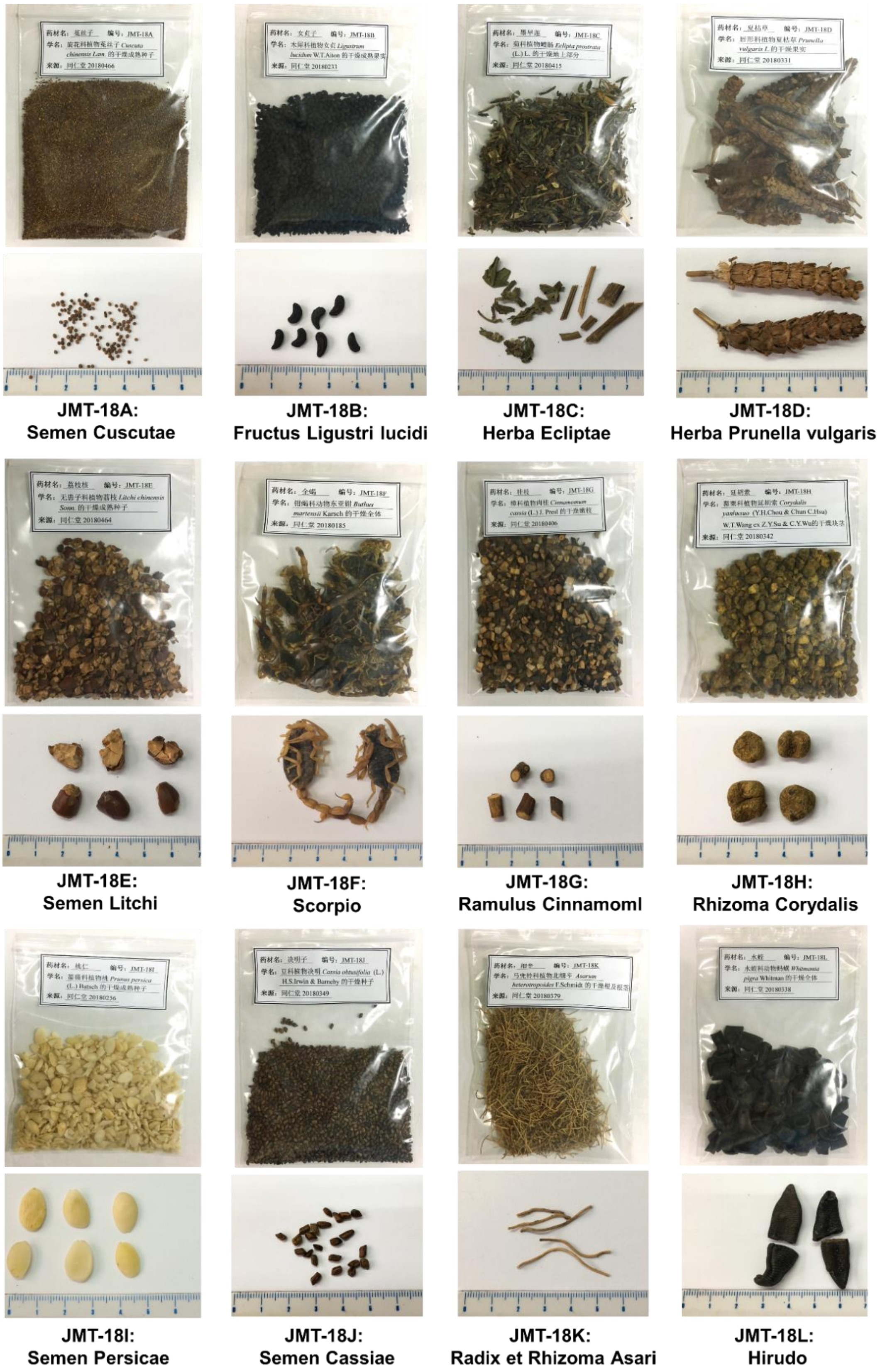

SUPPLEMENTARY FIGURE 1 | Scan of the vouchers for 12 kinds of crude drugs in Jin-Mai-Tong decoction (JMT). Botanic samples, JMT-A—JMT-E and JTM-G—JMT-K; animal samples, JMT-F and JMT-L.

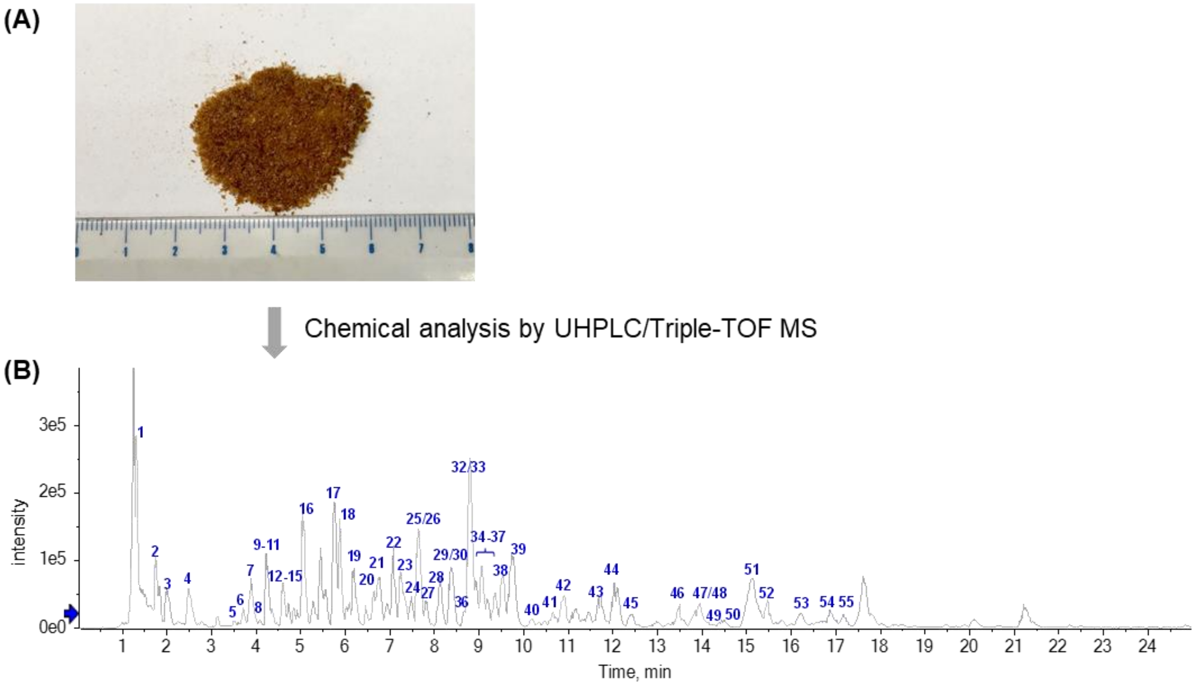

SUPPLEMENTARY FIGURE 2 | The freeze-dried powder of Jin-Mai-Tong decoction (JMT) (A) and the base peak chromatogram of JMT in ESI (-) ion mode (B).

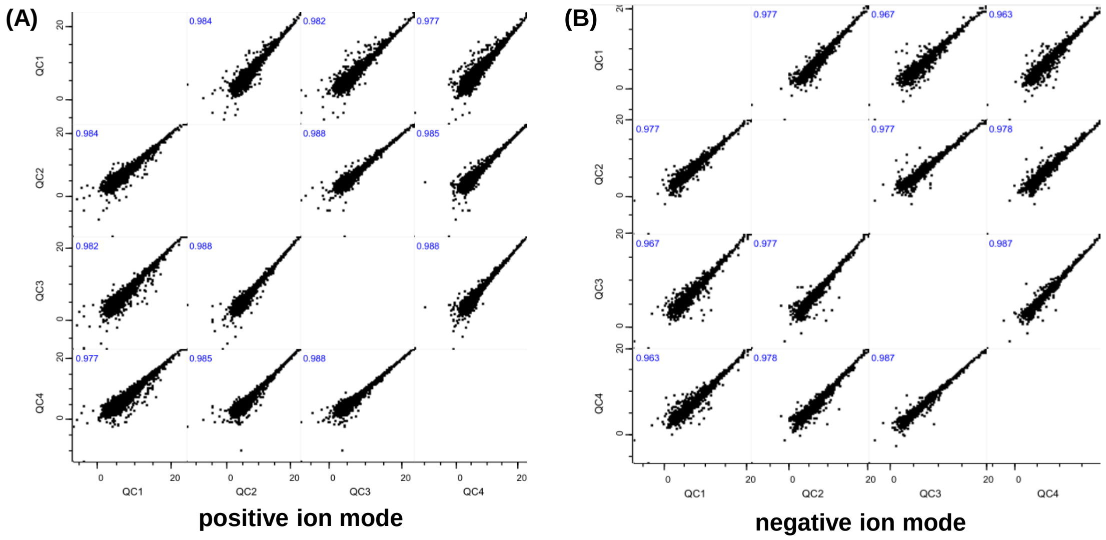

SUPPLEMENTARY FIGURE 3 | Correlation analysis of the QC samples in positive (A) and negative (B) ion modes. The values of r^2 were exhibited in blue font.

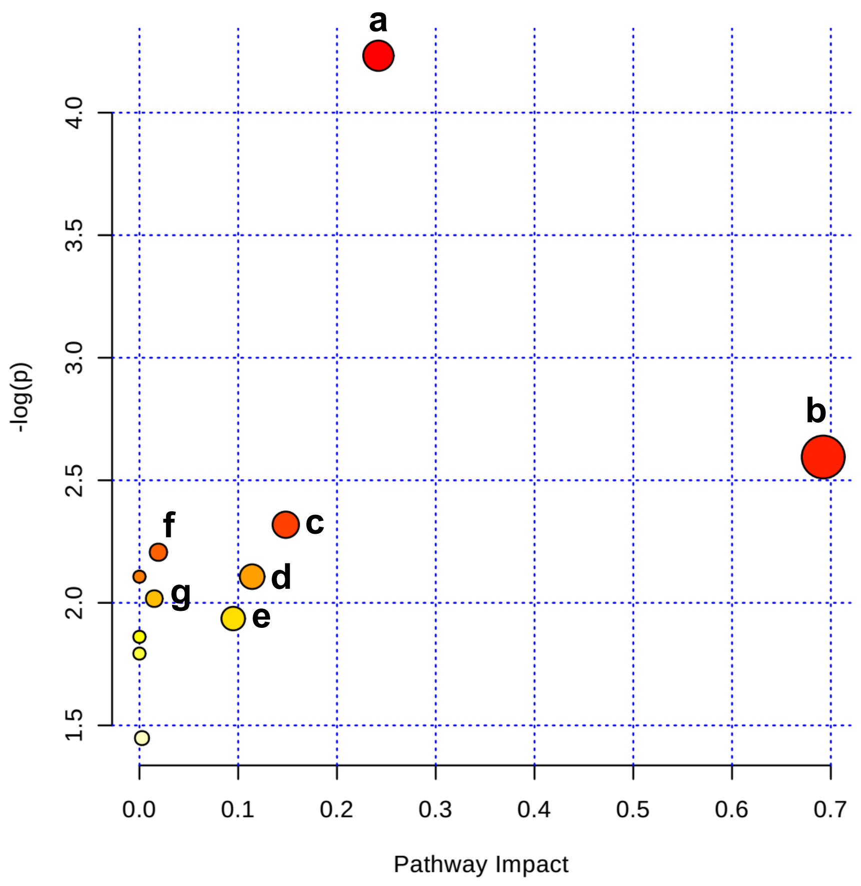

SUPPLEMENTARY FIGURE 4 | Bubble map from pathway Analysis. (a, glycerophospholipid metabolism; b, caffeine metabolism; c, glyoxylate and dicarboxylate metabolism; d, citrate cycle (TCA cycle); e, alanine, aspartate and glutamate metabolism; f, glycerolipid metabolism; g, pyruvate metabolism.

SUPPLEMENTARY TABLE 1 | Detailed information of the crude drug materials of Jin-Mai-Tong decoction (JMT) used in this study.

Voucher number	Drug name	Biological origins	Chinese name	Batch number
JMT-18A	Semen Cuscutae	the seeds of *Cuscuta chinensis* Lam.	Tu-Si-Zi	20180466
JMT-18B	Fructus Ligustri Iucidi	the seeds of *Ligustrum lucidum* W.T.Aiton	Nv-Zhen-Zi	20180233
JMT-18C	Herba Ecliptae	the herb of *Eclipta prostrata* (L.) L.	Mo-Han-Lian	20180451
JMT-18D	Herba Prunella Vulgaris	the herb of *Prunella vulgaris* L.	Xia-Ku-Cao	20180331
JMT-18E	Semen Litchi	the seeds of *Litchi chinensis* Sonn.	Li-Zhi-He	20180464
JMT-18F	Scorpio	the whole body of *Buthus martensii* Karsch	Quan-Xie	20180185
JMT-18G	Ramulus Cinnamoml	the tender stem of *Cinnamomum cassia* (L.) J. Presl	Gui-Zhi	20180406
JMT-18H	Rhizoma Corydalis	the rhizoma of *Corydalis yanhusuo* (Y.H.Chou & Chun C.Hsu) W.T.Wang ex Z.Y.Su & C.Y.Wu	Yan-Hu-Suo	20180342
JMT-18I	Semen Persicae	the seeds of *Prunus persica* (L.) Batsch	Tao-Ren	20180256
JMT-18J	Senmen Cassiae	the seeds of *Cassia obtusifolia* L.	Jue-Ming-Zi	20180349
JMT-18K	Radix et Rhizoma Asari	the radix and rhizoma of *Asarum heterotropoides* F.Schmidt	Xi-Xin	20180379
JMT-18L	Hirudo	the whole body of *Whitmania pigra* Whitman.	Shui-Zhi	20180338

SUPPLEMENTARY TABLE 2 | Compounds putatively identified from Jin-Mai-Tong decoction (JMT) by HPLC/Triple-TOF MS analysis

Peak No.	t_R (min)	Measured $[M-H]^-$ (m/z)	Predicted $[M-H]^-$ (m/z)	Δ (ppm)	Formula	(–) MS/MS (m/z)	Identification	Derived From
1	1.31	515.1158	515.1190	-6.21	$C_{25}H_{24}O_{12}$	353.1019, 191.0058	isochlorogenic acid C	C
2	1.75	167.0350	167.0342	4.79	$C_8H_8O_4$	135.0816, 133.0785	vanillic acid	D
3	2.15	153.0192	153.0188	2.61	$C_7H_6O_4$	109.0279	protocaechuic acid	E
4	2.50	353.1013	353.1025	-3.40	$C_{20}H_{18}O_6$	339.0871,	asarinin	J
5	3.51	633.4045	633.4003	6.63	$C_{36}H_{58}O_9$	471.3483, 453.3337	ecliptasaponin A or D	C
6	3.72	633.4055	633.4003	8.21	$C_{36}H_{58}O_9$	471.3481, 453.3340	ecliptasaponin A or D	C
7	3.93	289.0739	289.0712	9.34	$C_{15}H_{14}O_6$	245.0812, 205.0503	epicatichin	E
8	4.12	919.2774	919.2719	5.98	$C_{39}H_{52}O_{25}$	919.2722, 271.0615	cassiaside B2	I
9	4.31	685.2331	685.2344	-1.90	$C_{31}H_{42}O_{17}$	453.3316	specnuezhenide	B
10	4.43	313.0359	313.0348	3.51	$C_{16}H_{10}O_7$	297.1523	wedelolactone	C
11	4.50	609.1860	609.1819	6.73	$C_{28}H_{34}O_{15}$	447.1278, 285.0761	hesperidin	E
12	4.71	353.0871	353.0873	-0.57	$C_{16}H_{18}O_9$	191.0561	chlorogenic acid	A,C
13	4.79	957.5067	957.5059	0.84	$C_{48}H_{78}O_{19}$	795.4531, 455.3455	ecliptasaponin B or III	C
14	4.92	563.1431	563.1401	5.33	$C_{26}H_{28}O_{14}$	473.1090, 383.0771	schaftoside	E
15	4.96	340.1540	340.1549	-2.65	$C_{20}H_{23}NO_4$	178.056	tetrahydrojatrorrhizine	G
16	5.05	595.1705	595.1663	7.06	$C_{27}H_{32}O_{15}$	271.0633, 255.0393	rubrofusarin-6-o-β-gentiobioside	I
17	5.75	463.0896	463.0877	4.10	$C_{21}H_{20}O_{12}$	303.0428	hyperoside	A
18	5.88	419.1019	419.0978	9.78	$C_{20}H_{20}O_{10}$	257.0479	cassiaside	I
19	6.26	463.0901	463.0877	5.18	$C_{21}H_{20}O_{12}$	301.0344	isoquercitrin	A,C,D
20	6.68	253.0513	253.0501	4.74	$C_{15}H_{10}O_4$	225.0105	chrysophanol	C
21	6.85	491.1231	491.1190	8.35	$C_{23}H_{24}O_{12}$	329.0598	hesperidin-6-o-β-D-glucoside	I
22	7.05	447.0897	447.0927	-6.71	$C_{21}H_{20}O_{11}$	285.0425, 133.0292	luteoline 7Oglucoside	A,D

23	7.33	465.1032	465.1033	-0.22	$C_{21}H_{22}O_{12}$	285.0453	dihydroquercetin	B
24	7.51	795.4469	795.4531	-7.79	$C_{42}H_{68}O_{14}$	453.3356	ecliptasaponin I,C,IV or XV	C
25	7.63	447.0950	447.0927	5.14	$C_{21}H_{20}O_{11}$	285.0402, 151.0030	luteoloside	I
26	7.69	445.0751	445.0771	-4.49	$C_{21}H_{18}O_{11}$	269.0453	apigenin-7-O-glucronide	J
27	7.85	447.0939	447.0927	2.68	$C_{21}H_{20}O_{11}$	285.0395	astragalin	A
28	8.20	685.2359	685.2344	2.19	$C_{31}H_{42}O_{17}$	453.3308	nuezhenoside	B
29	8.41	785.2501	785.2504	-0.38	$C_{35}H_{46}O_{20}$	623.1962, 477.1308	echinacoside	B
30	8.50	843.4395	843.4378	2.02	$C_{42}H_{68}O_{17}$	633.3495, 471.3387	ecliptasaponin VI	C
31	8.69	359.0777	359.0767	2.78	$C_{18}H_{16}O_8$	322.0093	rosmarinic acid	D
32	8.80	299.1157	299.1131	8.69	$C_{14}H_{20}O_7$	137.0591	salidroside	B
33	8.95	1071.3583	1071.3557	2.43	$C_{48}H_{64}O_{27}$	1117.2850, 909.2386	oleonuezhenide	B
34	9.05	453.3329	453.3369	-8.82	$C_{30}H_{46}O_3$	437.3395	3-hydroxy oleanolic acid	C
35	9.21	301.0352	301.0348	1.33	$C_{15}H_{10}O_7$	165.9889	quercetin	A,C,D
36	9.35	505.1325	505.1346	-4.16	$C_{24}H_{26}O_{12}$	343.0828	cassiin	I
37	9.43	449.1102	449.1084	4.01	$C_{21}H_{22}O_{11}$	287.0543	eriodictyol-7-o-glucoside	J
38	9.60	451.1096	451.1088	1.77	$C_{17}H_{24}O_{14}$	407.2266	privet acid	B
39	9.74	368.1875	368.1862	3.53	$C_{22}H_{27}NO_4$	336.1039, 294.0850	corydaline	G
40	10.19	279.2311	279.2324	-4.66	$C_{18}H_{32}O_2$	279.2308	linoleic acid	H
41	10.69	283.0629	283.0607	7.77	$C_{16}H_{12}O_5$	268.0367	obtusifolin	I
42	10.95	285.0385	285.0399	-4.91	$C_{15}H_{10}O_6$	175.0387, 151.0006	kaempferol	A
43	11.81	431.1014	431.1009	1.16	$C_{21}H_{19}O_{10}$	269.0453	emodin-6-o-β-D-glucoside	I
44	12.05	343.0829	343.0818	3.21	$C_{18}H_{16}O_7$	285.0364	obtusin	I
45	12.47	357.1003	357.0975	7.84	$C_{19}H_{18}O_7$	313.0272, 269.0104	methyl Obtusin	I
46	13.50	329.0677	329.0661	4.86	$C_{17}H_{14}O_7$	270.0162	aurantio-obtusin	I
47	13.83	461.0710	461.0720	-2.17	$C_{21}H_{18}O_{12}$	285.0407,	scutellarin	J
48	14.00	519.1523	519.1503	3.85	$C_{25}H_{28}O_{12}$	227.1259	6'-O-cinnamoyl-8-epikingisidic acid	B

49	14.40	149.0605	149.0597	5.37	$C_9H_8O_2$	131.0486	cinnamic acid	F
50	14.55	461.0758	461.0720	8.24	$C_{21}H_{18}O_{12}$	279.0410, 285.0038	kaempferol-3-O-glucuronide	A
51	15.29	285.0413	285.0399	4.91	$C_{15}H_{10}O_6$	151.0041, 133.0256	luteolin	A,C
52	15.50	269.04561	269.0450	2.27	$C_{15}H_{10}O_5$	151.0033	apigenin	A
53	16.18	539.1820	539.1765	10.20	$C_{25}H_{32}O_{13}$	275.0839	oleuropein	B
54	16.90	287.0562	287.0556	2.09	$C_{15}H_{12}O_6$	151.0038	eriodictyol	J
55	17.17	255.2347	255.2324	9.01	$C_{16}H_{32}O_2$	255.2337	hexadecanoic acid	H

Note: A, Semen Cuscutae; B, glossy privet fruit; C, Eclipta prostrata; D, Prunella vulgaris; E, semen litchi; F, cassia twig; G,Corydalis Rhizoma; H, Persicae semen; I, Cassiae Semen; J, Asarum L.

SUPPLEMENTARY TABLE S3 | The precision of peak area for ten selected ions in positive and negative ion modes

ion mode	extracted ion (t_R_m/z)	QC-1	QC-2	QC-3	QC-4	RSD (%)
positive	0.73_407.9660	681	620	618	653	**4.67**
	4.16_503.2578	102040	106455	110882	116411	**5.64**
	8.54_255.2217	4508	4278	4406	4519	**2.53**
	12.11_889.9594	6308	6624	6813	6712	**3.30**
	16.20_743.5491	129487	122789	128084	109392	**7.48**
negative	0.55_143.0938	1617	1566	1461	1341	**8.17**
	4.76_671.2985	4056	3610	3338	3254	**10.13**
	8.53_301.2971	9040	8028	7357	7028	**11.29**
	12.75_629.8071	12546	11617	10731	10174	**9.22**
	16.89_955.4596	28305	22554	26636	28888	**10.75**
